# Conscientiousness protects visual search performance from the impact of fatigue

**DOI:** 10.1186/s41235-022-00410-9

**Published:** 2022-06-28

**Authors:** Justin N. Grady, Patrick H. Cox, Samoni Nag, Stephen R. Mitroff

**Affiliations:** 1 Department of Psychological and Brain Sciences, The George Washington University, 2125 G St NW, Washington, DC 20052 USA; 2 Intelligence Community Postdoctoral Research Fellowship Program, Department of Psychological and Brain Sciences, The George Washington University, Washington, DC 20052 USA

**Keywords:** Visual search, Individual differences, Fatigue, Conscientiousness, Personnel selection, Energy

## Abstract

Visual search—looking for targets among distractors—underlies many critical professions (e.g., radiology, aviation security) that demand optimal performance. As such, it is important to identify, understand, and ameliorate negative factors such as fatigue—mental and/or physical tiredness that leads to diminished function. One way to reduce the detrimental effects is to minimize fatigue itself (e.g., scheduled breaks, adjusting pre-shift behaviors), but this is not always possible or sufficient. The current study explored whether some individuals are less susceptible to the impact of fatigue than others; specifically, if conscientiousness, the ability to control impulses and plan, moderates fatigue’s impact. Participants (*N* = 374) self-reported their energy (i.e., the inverse of fatigue) and conscientiousness levels and completed a search task. Self-report measures were gathered prior to completing the search task as part of a large set of surveys so that participants could not anticipate any particular research question. Preregistered linear mixed-effect analyses revealed main effects of energy level (lower state energy related to lower accuracy) and conscientiousness (more trait conscientiousness related to higher accuracy), and, critically, a significant interaction between energy level and conscientiousness. A follow-up analysis, that was designed to illustrate the nature of the primary result, divided participants into above- vs. below-median conscientiousness groups and revealed a significant negative relationship between energy level and accuracy for the below median, but not above-median, group. The results raise intriguing operational possibilities for visual search professions, with the most direct implication being the incorporation of conscientiousness measures to personnel selection processes.

## Introduction

Visual search, looking for targets among distractors, is an important skill involving an array of underlying cognitive mechanisms, including perception, memory, attention, and decision making (Eckstein, 2011; Nakayama & Martini, 2011). Search is central to many aspects of normal everyday life (e.g., finding a friend in a crowd) and is also important for a wide array of specialized tasks (e.g., soccer goalkeepers scanning the field for opposing players making runs toward the goal; Savelsbergh et al., 2002). Visual search also underlies many professional tasks that can have life-or-death outcomes, including aviation security (e.g., Mitroff et al., 2018; Wetter, 2013), medical image perception (e.g., Horowitz, 2017; Krupinski, 2015; Van der Gijp et al., 2017), lifeguarding (e.g., Lanagan-Leitzel et al., 2015), and many military operations (e.g., Nelson et al., 2015). In such professions, human operators conduct what can be complicated tasks that demand high levels of attention, vigilance, and engagement (Krupinski, 2015; Wetters, 2013), but unfortunately, the operators can be susceptible to fatigue—a state of tiredness and diminished functioning.

Extensive research has explored the detrimental impact of fatigue on cognitive performance and occupational safety, suggesting that fatigue arises from an array of conditions, including long work hours, unusual shift times, and stressful employment settings, which are all common to professions involving visual search (e.g., Williamson & Friswell, 2013). Fatigue is broadly considered to be a complex multidimensional symptom in which individuals experience physical tiredness and a lack of energy (Schwid et al, 2003). Accordingly, fatigue has an array of implications for a range of cognitive and motor tasks and produces known deficits in areas such as sports performance (Smith et al., 2016), driving (Lal & Craig, 2001), and military operations (Miller et al., 2018).

For visual search, detriments from fatigue are generally thought to stem from both a prolonged time spent on a task and the searchers’ mental and/or physical state when starting the task (Bailey et al., 2007). While both factors are important, the current study focused on participants’ mental and/or physical state when they began a testing session (i.e., “fitness-for-duty”). In professional visual search environments, each operator arrives at work in their own particular state of readiness, which can vary between and within individuals from one day to the next. Since employers cannot control or mandate how their employees spend their time before starting their shift or the length and quality of their sleep, it is especially important to understand the potential relationships between fitness-for-duty related fatigue and performance. Past research has explored such fitness-for-duty issues, with a focus on how an individual’s state before engaging with an important task (e.g., working in a nuclear power plant, serving military or law enforcement duties, driving long distances) can affect success (e.g., Baas et al., 2000; Bendak & Rashid, 2020).

Prior in-lab research has established an effect of fatigue on visual search performance. For example, sleep deprivation (one form of pre-task fatigue) has been shown to impact both accuracy and response time on visual search tasks (Santhi et al., 2007). Other studies found that sleep-related fatigue impacted visual search response time, but not accuracy (De Gennaro et al., 2001; Pomplun et al., 2012). More broadly, fatigued individuals generally struggle to maintain both fast response times and high accuracy, which can lead to a speed-accuracy trade-off wherein individuals either hold accuracy high by slowing their response time or maintain quick response times at the expense of accuracy depending on the parameters of the task. Given the emphasis of speed and precision (i.e., efficiency) in many real-world searches, it is vital that operators are both highly accurate and quick to respond.

Academic radiology and other applied research fields have consistently demonstrated an impact of fatigue on performance (e.g., Krupinski, 2015; Taylor-Phillips & Stinton, 2019; Vosshenrich et al., 2021). For example, research has shown that radiologists’ performance varies over the course of the day, generally peaking in the early evening, but suffering in the middle of the day following lunch (Monk, 2005). This “post-lunch dip effect” corresponds to a well-studied period of postprandial fatigue after the midday meal (Stahl et al., 1983). Similarly, research has shown that professional radiologists exhibit within-subject performance decrements following overnight shifts; diagnostic performance was worse and subjective self-reports of fatigue were higher when assessed the morning after an overnight shift compared to after a normal day shift (Hanna et al., 2018). Such effects of fatigue can have profound implications for radiology, as subpar visual search performance could lead to missed abnormalities (Krupinski, 2015).

The above findings highlight a robust impact of fatigue that represents a serious concern for the successful execution of visual search in critical environments, but does this impact vary across individuals? Prior research suggests that it might. For example, a study that looked at the impact of sleep deprivation with fighter pilots and non-pilots (Caldwell et al., 2005) found both individual- and group-level differences in the susceptibility to fatigue. At the individual level, one of the pilots showed no effect of sleep deprivation after 37-h of wakefulness, while others showed steep drops in performance. At the group level, the pilots collectively showed greater resilience, with their cortical activation data being more similar to fatigue-resistant non-pilots than fatigue-vulnerable non-pilots (Caldwell et al., 2005). This is just one example, but it highlights that it may be possible to leverage individual differences in resilience to fatigue to better understand the mechanisms by which fatigue affects cognition and to inform operational practices such as hiring decisions.

The current study focused on one particular factor of individual differences that might moderate the impact of fatigue on visual search performance: conscientiousness—the ability to control impulses, be goal directed, plan, and delay gratification (Roberts et al., 2009). Conscientiousness is a strong candidate trait to study here as a possible individual difference moderator on the impact of fatigue given that previous work has shown that more conscientious individuals are better searchers (Biggs et al., 2017; Spain et al., 2017). Moreover, conscientiousness may facilitate core cognitive abilities that underlie visual search performance. For example, it has been suggested that more conscientious individuals place a higher degree of emphasis on success and rule learning compared to less conscientious individuals, leading to higher accuracy on working memory tasks (Studer-Luethi et al., 2012) and superior performance on cognitive shifting tasks (Fleming et al., 2016). Further, previous research suggests that conscientiousness may act as a protective factor against fatigue; those with higher levels of conscientiousness were more resistant to dangerous microsleep episodes while driving after a period of sleep deprivation, compared to their less conscientious counterparts (Hidalgo-Gadea et al., 2021). Collectively, the prior work on fatigue and conscientiousness presents the intriguing possibility that conscientiousness may serve as a meaningful factor that could enhance, or diminish, the negative impacts of fatigue on visual search.

The goal of the current study was to explore if the trait factor of conscientiousness moderates the relationship between the state factor of fatigue and visual search performance. An individual differences approach was used wherein participants completed a large set of self-report surveys and a visual search task, and the primary question was whether self-reported conscientiousness (measured via the Big-5 Inventory) would significantly moderate the relationship between energy level (i.e., the inverse of fatigue) and visual search performance.

## Materials and methods

The study design and analyses were preregistered (https://osf.io/7w8dm). Specifically, while the data were collected as part of a broader research effort conducted in the laboratory over four years, the participant inclusion/exclusion steps (described below) and the primary analyses were preregistered before being executed. Two exploratory follow-up analyses were not preregistered, and they were conducted to illustrate the direction of the effort and provide visualizations (Fig. 3). This research complied with the American Psychological Association Code of Ethics and was approved by the Institutional Review Board at The George Washington University (GWU). Informed consent was obtained from each participant. The data for the current study were drawn from a larger project conducted in the GWU Visual Cognition Lab wherein participants responded to self-report surveys and participated in behavioral tasks (see Appendix). Data from this project have been used for other purposes (e.g., Nadler et al., 2021; Silverman et al., 2022), and the current study only analyzed the specific subset of the data reported here; while there were hundreds of possible questions that could be explored with the variety of surveys administered (see Appendix), the current project involved preregistering a very specific, constrained set of questions to examine. All eligible participants (see below for exclusion criteria) were included in the analyses.

### Participants

Participants (*N* = 578) were recruited from November 2016 to April 2021 through the GWU Department of Psychological & Brain Sciences’ subject pool and received course credit. Data collection efforts and timing were constrained by the University’s semester schedule and other external influences (e.g., the COVID-19 pandemic). Enrolled participants could withdraw without penalty and could skip specific self-report questions. A final dataset was determined via the below ordered series of preregistered, sequential data exclusion steps.

#### Non-performance-based data exclusion: 578 to 559 participants

The first data exclusion step focused on non-performance-based criteria, removing data from participants who self-reported to being outside the age range of 18 to 25 (*N* = 15) or who completed the study twice (second datasets removed, *N* = 4). The preregistered age range was selected as it encompassed the vast majority of the participant pool and age effects were not a focus of the current project.

#### Self-report survey performance-based data exclusion: 559 to 528 participants

The second data exclusion phase removed data in three sequential steps. First, three “attention check” questions were sporadically built into the self-report surveys (see Procedures) to ensure participants read and comprehended the survey prompts. The attention checks were simple multiple-choice questions about the upcoming survey and provided feedback for wrong answers. If participants required four attempts on any one check or required seven or more (out of a possible 12) total attempts across all three checks, their full dataset was removed from all analyses (*N* = 22). Second, ten of the surveys (Appendix surveys #4–5, 9–10, 13–18) were selected, and if a participant did not respond to at least half of the questions for three or more of the surveys, then their data were removed from all analyses (*N* = 1). Third, for the same subset of ten surveys, participants’ responses were assessed to determine if they had no variability across the entire survey. Participants’ data were removed from all analyses if they clicked the same radio button for the entire survey for three or more surveys (*N* = 8).

#### Behavioral visual search task data exclusion: 528 to 376 participants

Of the remaining 528 participants with usable self-report survey data, 397 also completed the behavioral visual search task. Participants’ data were subsequently excluded if they failed the minimum performance-based criteria; in sequential order, data were removed if the participants did not complete exactly 72 trials of the *Big Plane Challenge* level (*N* = 12), if they had a target-present accuracy less than 20% (*N* = 6), or if they had a false alarm rate greater than 80% (*N* = 3).

#### Survey-specific exclusion: 376 to 374 participants

A final exclusion step removed data from participants who did not have a complete dataset for the three measures of interest for the current project: energy level (i.e., the inverse of fatigue), conscientiousness, and visual search performance. Data were removed from one participant for not responding to the energy level question and one participant for not providing sufficient data for the conscientiousness measure (the participant did not answer at least half of the questions in the Big-5 Personality Inventory survey)—leaving a final dataset of 374 participants.

#### Participant cohorts

Participants from November 2016 to March 2020 were tested in the GWU Visual Cognition Lab in person (“in-lab” cohort; *N* = 205, mean age = 19.69 years, SD = 1.19 years, 165 female, 39 male, 1 did not report gender), and participants from April 2020 to April 2021 were tested virtually using their own computer and mobile device technology (“virtual” cohort; *N* = 169, mean age = 19.99 years, SD = 1.36 years, 105 female, 62 male, 2 did not report gender). While there was no a priori reason to predict that these two cohorts would show different effects for the primary measures of interest, prior work has shown that the cohorts differ in general (Nadler et al., 2021). As such, cohort (in-lab vs virtual) was preregistered as a factor of no interest to be included in the planned statistical analyses.

### Procedure

#### Self-report survey data

**Fig. 1.**
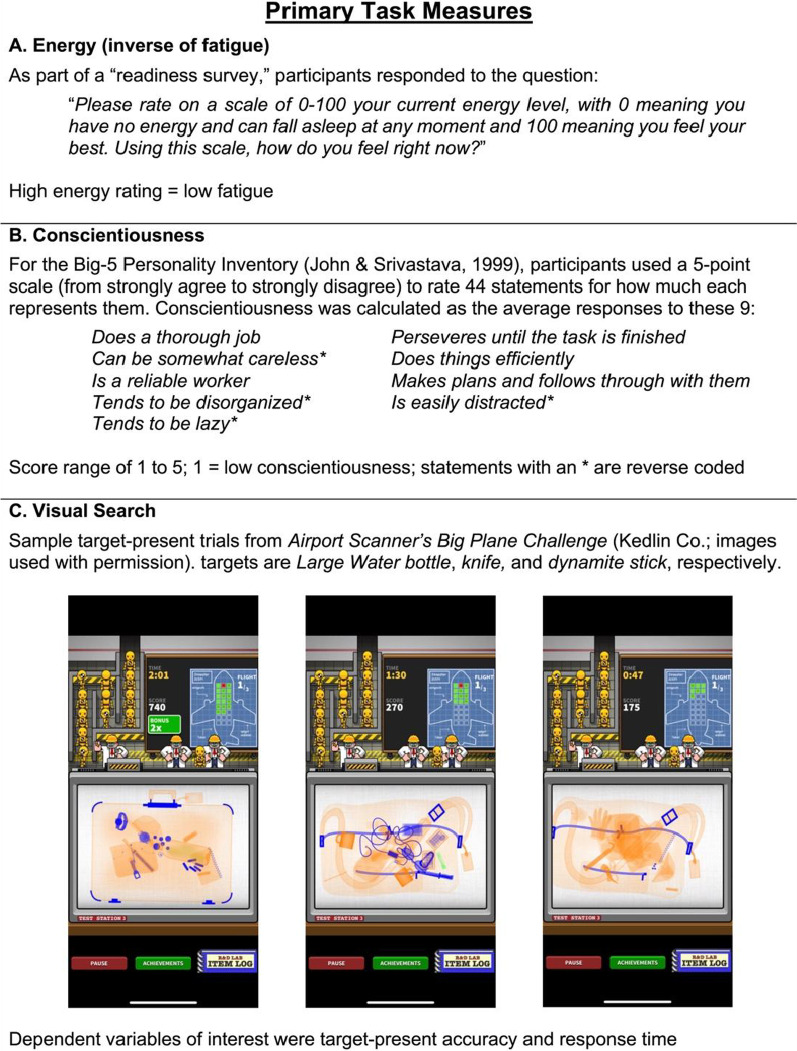
Study measures of **A** energy (i.e., the inverse of fatigue), **B** conscientiousness, and **C** visual search

Participants completed a large set of self-report surveys (see Appendix) via Qualtrics, but only two were assessed for the current study. First, participants completed a “readiness survey” that asked about typical and recent sleeping habits, caffeine use, and other related factors. One question asked participants to rate their energy level on a scale of 0–100 (Fig. 1A), with lower energy level ratings corresponding to higher fatigue and vice versa. This self-reported measure of energy served as the operational definition of fatigue for the current project. Self-report measures of energy are commonly used (e.g., De Gennaro et al., 2001; LaChapelle & Finlayson, 1998); for example, on the Stanford Sleepiness Scale (Hoddes et al., 1973) and the Visuo-analogue Sleepiness Scale (Fransson et al., 2008) participants rate how they feel within a certain range (e.g., marking how sleepy they are from “not at all” to “very much”). Prior research supports the use of such subjective reports given that individuals have insight into their own current state of fatigue—self-report measures of fatigue have been shown to directly link to objective sleepiness as measured by EEG (Åkerstedt & Gillberg, 1990).

The second measure of interest was the conscientiousness subscale from the Big-5 Personality Inventory (John & Srivastava, 1999). This subscale, which is calculated as the average response on a 1 to 5 scale for 9 questions, was used to measure participants’ self-reported level of conscientiousness (Fig. 1B). No other survey data, including other subscales from the Big-5 Inventory, were analyzed as part of this project as laid out in the preregistered analysis plans.

#### Behavioral visual search task

The visual search performance measures came from *Airport Scanner* (Kedlin Co.)—a mobile game wherein players search for prohibited items in simulated bags at a virtual airport security checkpoint (Fig. 1C). *Airport Scanner* was a publicly available game and the developers made the data available for research purposes (e.g., Ericson et al., 2017; Kramer et al., 2022; Mitroff et al., 2015). Participants from the in-lab cohort completed the *Airport Scanner* gameplay on an iPad tablet provided to them in the testing room. The participants in the virtual cohort completed the gameplay on their own devices in their own environment.

All behavioral data for the current study came from the *Big Plane Challenge* in the *R&D Lab* level of the game (e.g., Mitroff et al., 2018). After completing two tutorial levels, 12 and 24 trials, respectively, that provided practice and introduced the game dynamics, players completed 72 trials of gameplay. A trial was defined as a single bag that moved laterally across the screen. If participants detected a prohibited item (i.e., target), they were instructed to use their finger to tap on the screen at the location of the item. If the bag did not contain any prohibited items (~ 50% of the trials), participants could either swipe the bag across the screen or let it move through the scanner by itself. Approximately 50% of the trials contained a single target (drawn from a set of 20 possible items), and each trial contained 5 to 15 distractor items (drawn, with replacement, from a set of 100 possible items). Participants were presented with a timer measuring total time elapsed and a score based on successful trials. Trial-level data were removed if the response time was quicker than 250 ms or longer than 10 s (21 out of 26,928 total trials removed; 17 participants had 1 trial removed and 2 participants had 2).

#### Planned analyses

As preregistered, linear mixed-effect (LME) models were used to assess target-present accuracy and target-present response time as dependent variables with energy level, conscientiousness, and their interaction as the fixed effects of interest. Gender (male, female, no response) and participant cohort (in-lab, virtual) were assessed as categorical random effects of no interest. The LME models were defined as: *accuracy [or response time]* ~ *1* + *energy level* + *conscientiousness* + energy level*conscientiousness + *(1|gender)* + *(1|cohort)*. The interaction effect of energy level and conscientiousness for each model was of primary interest, given the hypothesis that the effect of energy (inverse of fatigue) on visual search performance would be moderated by conscientiousness. Effect sizes were calculated for each fixed effect term of interest in the LME model as Cohen’s *f *^*2*^, which represents the ratio of the unique variance explained by a given term in the model to the unexplained variance (Selya et al., 2012).

## Results

### Descriptive results

The average energy level rating was 62.56 (SD = 19.29, range = 10–100), and the average conscientiousness score was 3.55 (SD = 0.58, range = 2.22–5). The average visual search target-present accuracy was 71.56% (SD = 14.13%, range = 21.05–97.37%), and the average visual search target-present response time was 2713.56 ms (SD = 502.00 ms, range = 1574.74–4459.63 ms).

### Primary analysis: Preregistered Linear mixed-effect (LME) models

**Fig. 2.**
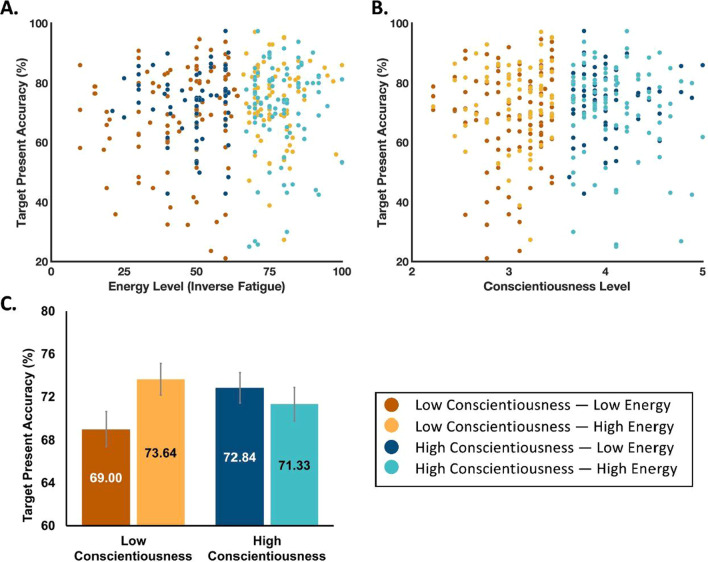
Target-present accuracy by energy and conscientiousness to provide an illustration of the LME model of accuracy. All data are grouped relative to median energy level and median conscientiousness as indicated in the legend in the lower right. **A** Accuracy by energy. **B** Accuracy by conscientiousness level. **C **Data divided into four groups to illustrate the primary linear mixed-effect model analysis on accuracy. Error bars represent standard error

The LME model for target-present accuracy produced a significant main effect of the energy level [*F*(1, 370) = 5.071; *p* = 0.025; *f*^2^ = 0.0137] such that higher levels of energy were related to higher accuracy (i.e., fatigue, the inverse of energy, was negatively correlated with accuracy, Fig. 2A), and a main effect of conscientiousness [*F*(1, 370) = 4.516; *p* = 0.034; *f*^2^ = 0.0122] such that higher levels of conscientiousness were related to higher accuracy (i.e., conscientiousness was positively correlated with accuracy, Fig. 2B). Most critical for the current project, the model produced a significant interaction between energy and conscientiousness on visual search accuracy [*F*(1, 370) = 4.117; *p* = 0.043; *f*^2^ = 0.0111]. The LME model for target-present response time produced no significant main effects [energy level: *F(1, 370)* = 2.312*; p* = 0.129; *f*^2^ = 0.0061]; conscientiousness: [*F(1, 370)* = 2.486*; p* = 0.116; *f*^2^ = 0.0066), nor a significant interaction [*F(1, 370)* = 1.993*; p* = 0.159; *f *^*2*^ = 0.0053].

**Fig. 3.**
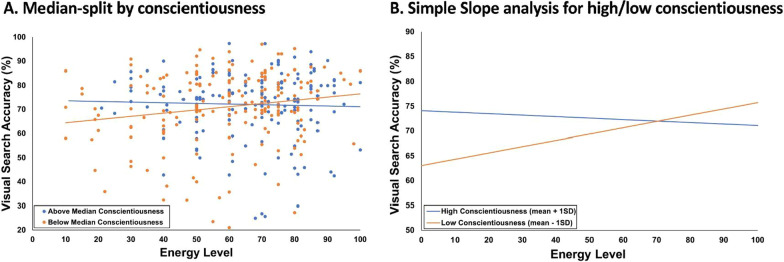
Non-preregistered analyses to illustrate nature of primary result: **A** Target-present accuracy by energy, separated for the participants with above-median (blue) and below-median (orange) conscientiousness scores. The vertical axis range begins at 20 given that data exclusion parameters removed visual search data from participants with an average performance below 20%. **B** Computed simple slope analysis for target-present accuracy by energy for data one standard deviation above (blue) and below (orange) the mean

Interpreting interaction effects requires visualization, and interpreting interaction effects between two continuous variables often entails grouping one or both of the continuous variables. Figure 2C depicts a grouping of the conscientiousness and energy level variables as high or low relative to the median. These median splits resulted in four groups: Low Conscientiousness—Low Energy, Low Conscientiousness—High Energy, High Conscientiousness—Low Energy, and High Conscientiousness—High Energy. The following section (and Fig. 3) provides further illustrations of the interaction effect from the LME model on accuracy.

### Follow-up analysis to illustrate nature of the moderation of the effect of energy level on target-present accuracy by conscientiousness

Given the significant interaction between energy level and conscientiousness in the LME model of accuracy, follow-up analyses that were not preregistered were conducted to further illustrate the nature of this relationship. For the first follow-up analysis, participants were divided into above-median and below-median conscientiousness groups based on a median split of the conscientiousness scores, and separate correlation analyses of visual search accuracy as a function of energy were conducted for each group (Fig. 3A). The correlations for each group were compared using Fisher's *r* to *z* transformation. The above-median conscientiousness group had an average conscientiousness score of 4.06 (SD = 0.34), and there was not a significant correlation between energy and accuracy (*N* = 169, *r* = −0.034, *p* = 0.661). The below-median conscientiousness group had an average conscientiousness score of 3.066 (SD = 0.31), and there was a significant correlation between energy and accuracy (*N* = 183, *r* = 0.176, *p* = 0.017), with higher energy corresponding to higher visual search accuracy. The two correlations significantly differed from one another; *z* = 1.97, *p* = 0.049. While this analysis was done for illustrative purposes and should not be over interpreted, the correlation values represent the effect size of the relationships; r-squared values of 0.001 for the above-median conscientiousness group, and 0.03 for the below-median conscientiousness group, suggest that effectively none of the variance in visual search accuracy is accounted for by self-reported fatigue for the above-median group, whereas roughly 3% of the variance in visual search accuracy is accounted for by fatigue for the below-median group.

For the second follow-up analysis, a simple slopes analysis was conducted, where the values of the moderating variables were fixed at points of interest (Aiken & West, 1991), and the resulting slopes were computed and statistically analyzed. For continuous variables without particular values of interest, it is common practice to use values of the mean ± one standard deviation (Fig. 3B). The high conscientiousness level (mean + 1 SD) of the simple slope analysis resulted in a non-significant slope of *β*_hi_ = −0.03; *t*(370) = −0.523; *p* = 0.601, whereas the low conscientiousness level (mean − 1 SD) of the simple slope analysis resulted in a significant slope of *β*_lo_ = 0.13; *t*(370) = 2.517; *p* = 0.012. Furthermore, a direct comparison of the simple slopes for the high level of conscientiousness vs. the low level of conscientiousness revealed a significant difference [*t*(370) = 2.904; *p* = 0.004].

## Discussion

The current study used an individual differences approach to explore the relationship between fatigue (operationally defined as the inverse of self-reported energy) and visual search performance. Fatigue negatively affects search (Bailey et al., 2007; De Gennaro et al., 2001; Santhi et al., 2007), which has profound implications for a wide swath of professions, including aviation security, radiology, lifeguarding, and more. Given that a mistake in such professions could have life-or-death consequences, it is important to know which stable factors may help to combat fatigue-induced performance decrements.

This study first contributed to the extant literature by providing supporting data that fatigue negatively related to visual search accuracy. Specifically, there was a main effect of energy level such that higher levels of energy corresponded to higher accuracy, which is in line with previous results (Hanna et al., 2018; Santhi et al., 2007). The current study focused on fatigue defined as the individuals’ state of readiness when beginning the task, demonstrating that those who start a visual search task fatigued are more likely to miss targets. Second, there was also a significant main effect of conscientiousness (higher levels of conscientiousness related to higher visual search accuracy), which supports prior results (Biggs et al., 2017; Spain et al., 2017). Finally, the primary question and novel finding of the current study was that the impact of fatigue on search accuracy was moderated by conscientiousness. Individuals who self-reported higher levels of conscientiousness did not demonstrate a significant effect of energy level on accuracy, while those who self-reported lower levels of conscientiousness did, with lower energy relating to lower accuracy (Fig. 2C).

There was no observed significant relationship between fatigue and response time, yet previous work has found such a link (De Gennaro et al., 2001). There are several possible explanations for this, including differences in the task design. For example, a speed-accuracy trade-off, where accuracy is held high at the expense of response time, is more commonly found in self-paced tasks when participants have unlimited time to initiate a response (Wilkinson, 1969). The dynamics of the visual search task employed here contained both self-paced and experimenter-paced aspects, making room for multiple possible outcomes.

As for many individual differences studies, it is important to understand and acknowledge potential unintended confounds that could limit or affect generalizability (Mitroff et al., under revision). For example, study designs and data exclusion criteria can inadvertently introduce sampling biases. In the current study, participants were asked to complete a large set of self-report surveys and behavioral tasks, which entailed volunteering for a relatively long experiment. Further, to be included in the analyses, participants had to meet specific inclusion criteria, including passing various attention checks and meeting specific performance checks on the behavioral task. It is likely that the experimental process created biases against individuals low in conscientiousness as low conscientious individuals may be less likely to sign up for a multiple-part study, fully complete the study, and/or pass the various inclusion steps. Moreover, the college-based sample explored here may be a more limited sample than what is represented in the broader public. Significant effects were nevertheless found, but it is possible that the study design could have worked against the hypothesis by restricting the full range of individual variability in conscientiousness.

The computed effect size estimates for the LME model were relatively small, which could arise for several reasons. For example, it should be expected that aspects of fatigue, conscientiousness, and their interaction should only be a small part of what determines each individual's performance. There is a myriad of individual differences factors that could be at play, and these aspects may contribute to the overall success for each individual, but it is reasonable to assume they are not a sole or overwhelming force. Further, given this is a university-based population, it is possible that the impact of fatigue and conscientiousness could be relatively dampened compared to a professional population. It is also notable that the data were not binned nor averaged for the primary LME analyses, which highlights that the effects, regardless of how large or small they might be, are statistically meaningful at the individual level.

### Implications for cognitive psychology literature

Individual differences have become an area of great interest for cognitive psychology research, and this study offers an example of gaining insight by accounting for a personality difference. It is worth noting that while conscientiousness was the sole stable individual difference measure assessed here, it is possible that other measures could also play a meaningful role. Likewise, the current study was cross-sectional, and it could be informative to implement a longitudinal study that assesses the same individuals multiple times. This is especially intriguing given that the primary result from the current study was that a trait-like factor (conscientiousness) impacted a state-like factor (fatigue); there might be mechanistically informative nuances revealed by exploring other trait–state relationships. Further, it would be exciting to explore whether training or intervention techniques could impact the relationship between fatigue and conscientiousness for search performance (e.g., could individuals be trained to become more conscientious and thereby more fatigue-resistant searchers?).

More broadly, it is worth considering the nature of the currently employed study procedures and how they might offer a roadmap for future academic research efforts. This study took advantage of an extensive data collection effort that tested a large cohort of participants on a wide range of assessments (Appendix). One key advantage of this process is that there was no indication to the participants of any particular experimental goal or hypothesis. In fact, the current experimental hypothesis was developed *after* the data collection effort. A potential downside of this process, however, is that a research team could conduct analyses to assess multiple hypotheses and then choose to only publish a subset, which could result in false discoveries (e.g., Kravitz & Mitroff, 2020). However, the critical step of preregistration alleviates this concern. The current study, for example, preregistered analyses and only assessed the subset of data a priori identified as of interest. This experimental procedure thus allows for gathering unbiased data that addresses a number of potential variables of interest, and then later preregistering specific research questions to explore. This process may prove quite useful, especially for individual differences research questions.

### Implications for visual search professions

The current findings have the potential to inform professional operations that rely on visual search. For example, knowing that more conscientious individuals are less susceptible to the negative impacts of fatigue could inform hiring and staffing decisions for aviation security, military operations, lifeguarding, and more. A fair question, though, is just how operationally meaningful is this particular effect; is the impact of the fatigue-conscientiousness relationship relevant for visual search professions, and should they consider incorporating this knowledge into their operational plans? Prior work using the same experimental paradigm provides insight to this issue. Specifically, in a prior study (Mitroff et al., 2018), US airport screening officers completed a visual search task that was nearly identical to the one used in the current study, and the professional security screeners completed a commercial version of the level of the publicly available game that was used in the current study. Critically, the officers’ performance in that task significantly correlated with their on-job performance such that those who were better at the visual search task were both more accurate and quicker at actual checkpoints. This suggests that this paradigm is sensitive to operational outcomes, and the current results demonstrate that this paradigm is also sensitive to the impacts of fatigue and conscientiousness. As such, it is reasonable to suggest that these data offer a potentially generalizable outcome with practical implications. Note that the participants who self-reported lower levels of conscientiousness varied in visual search accuracy by approximately 5% based upon their level of fatigue in Fig. 2C. This 5% difference is simply an average estimate based on splitting the data into above- and below-median groups, so it could be an over- or under-estimate. Nevertheless, even a minor shift in accuracy could manifest in a massive operational impact for aviation security given the sheer number of searches conducted each day across airports around the world and the implications of even a single missed target.

### Conclusions

The current project found that fatigue negatively impacted search performance, but that this relationship was significantly moderated by conscientiousness such that more conscientious individuals showed no relationship between fatigue and search accuracy. This exciting, but straightforward, result could have clear and direct implications for many professional settings that rely on visual search.

## Data Availability

The dataset and analysis code are available on the project’s OSF site (https://osf.io/8jzwc/).

## Appendix

The current study drew data from a broader research project that involved administering a large set of self-report surveys and behavioral measures.

### Self-report surveys

Participants completed the below self-report surveys as part of their participation in the project, but the only data analyzed in the current study were one question from the “readiness survey” (#2) and the conscientiousness measure from the Personality Inventory (#17).

1. Basic Demographics Survey (12 questions)
  - Survey constructed in GW Visual Cognition Lab.
2. Readiness Survey (14 questions)
  - Questions about sleep, caffeine use, energy, and related influences on readiness. Survey constructed in GW Visual Cognitive Lab.
3. Modified Edinburgh Handedness Inventory (13 questions)
  - Edlin, J. M., Leppanen, M. L., Fain, R. J., Hackländer, R. P., Hanaver-Torrez, S. D., & Lyle, K. B. (2015). On the use (and misuse?) of the Edinburgh Handedness Inventory. *Brain and Cognition, 94*, 44-51.
4. State-Trait Anxiety Inventory (40 questions)
  - Spielberger, C. D., Gorsuch, R. L., Lushene, R., Vagg, P. R., & Jacobs, G. A. (1983). Manual for the State-Trait Anxiety Inventory. Palo Alto, CA: Consulting Psychologists Press.
5. Adult ADD/ADHD Questionnaire (24 questions)
  - Jasper, L., & Goldberg, I. (1993). Jasper/Goldberg Adult ADD Questionnaire Retrieved October 1, 2009, from http://www.mentalhelp.net/poc/view_doc.php?id=974&type=doc&cn=ADHD
6. Concussion history (29 questions)
  - Survey constructed in GW Visual Cognition lab.
7. Hyperfocusing/flow survey (19 questions)
  - Csikszentmihalyi, Mihaly (Ed); Csikszentmihalyi, Isabella Selega (Ed). (1988). Optimal experience: Psychological studies of flow in consciousness (pp. 15–35). New York, NY, US: Cambridge University Press, xiv, 416 pp.
8. Video-game questionnaire (77 questions)
  - Green, C. S., Kattner, F., Eichenbaum, A., Bediou, B., Adams, D. M., Mayer, R. E., & Bavelier, D. (2017). Playing some video games but not others is related to cognitive abilities: A critique of Unsworth et al. (2015). *Psychological Science, 28*(5), 679-682. https://journals.sagepub.com/doi/suppl/10.1177/0956797616644837/suppl_file/Video-Game-Expertise_Classification_Scheme.pdf
9. Maximization Scale (13 questions)
  - Schwartz, B., Ward, A., Monterosso, J., Lyubomirsky, S., White, K., & Lehman, D. R. (2002). Maximizing versus satisficing: happiness is a matter of choice. *Journal of Personality and Social Psychology, 83*(5), 1178.
10. Barratt Impulsivity Scale (30 questions)
  - Patton, J. H., Stanford, M. S., & Barratt, E. S. (1995). Factor structure of the Barratt impulsiveness scale. [Research support, non-U.S. Gov’t]. *Journal of Clinical Psychology, 51*(6), 768–774.
11. Pittsburgh Sleep Quality Index (20 questions)
  - Buysse, D. J., Reynolds III, C. F., Monk, T. H., Berman, S. R., & Kupfer, D. J. (1989). The Pittsburgh Sleep Quality Index: A new instrument for psychiatric practice and research. *Psychiatry Research, 28*(2), 193–213.
12. Pastimes (18 questions)
  - Survey constructed in GW Visual Cognition Lab.
13. Autism-spectrum Quotient (50 questions)
  - Baron-Cohen, S., Wheelwright, S., Skinner, R., Martin, J., & Clubley, E. (2001). The autism-spectrum quotient (AQ): Evidence from asperger syndrome/high-functioning autism, males and females, scientists and mathematicians. *Journal of Autism and Developmental Disorders, 31*(1), 5–17.
14. Responsibility Attitudes Scale (26 questions)
  - Salkovskis, P. M., Wroe, A. L., Gledhill, A., Morrison, N., Forrester, E., Richards, C., ... & Thorpe, S. (2000). Responsibility attitudes and interpretations are characteristic of obsessive compulsive disorder. *Behaviour Research and Therapy, 38*(4), 347–372.
15. Obsessive–Compulsive Scale (20 questions)
  - Abramowitz, J. S., Deacon, B. J., Olatunji, B. O., Wheaton, M. G., Berman, N. C., Losardo, D., ... & Hale, L. R. (2010). Assessment of obsessive-compulsive symptom dimensions: development and evaluation of the Dimensional Obsessive-Compulsive Scale. *Psychological Assessment, 22*(1), 180.
16. Grit (10 questions)
  - Duckworth, A. L., Peterson, C., Matthews, M. D., & Kelly, D. R. (2007). Grit: perseverance and passion for long-term goals. *Journal of Personality and Social Psychology, 92*(6), 1087.
17. Personality Inventory—Big Five Inventory (44 questions)
  - John, O. P., & Srivastava, S. (1999). The Big-Five trait taxonomy: History, measurement, and theoretical perspectives (Vol. 2, pp. 102–138). Berkeley: University of California.
18. Self-control Scale (35 questions)
  - Tangney, J. P., Baumeister, R. F., & Boone, A. L. (2004). High self‐control predicts good adjustment, less pathology, better grades, and interpersonal success. *Journal of Personality, 72*(2), 271–324.
19. Media Multitasking Measure-Short (9 questions)
  - Baumgartner, S. E., Lemmens, J. S., Weeda, W. D., & Huizinga, M. (2017). Measuring media multitasking. Development of a short measure of media multitasking. *Journal of Media Psychology, 29*(2), 92–101.

### Behavioral tasks

Participants from the in-lab and virtual cohorts completed the *Airport Scanner* task described in the main text. Participants from the in-lab cohort also completed additional behavior tasks that were not analyzed in the current study:

1. Simple reaction time task
  - Participants made a speeded response to a visual stimulus to obtain a baseline measure of reaction timing.
2. Continuous performance task (AX-CPT)
  - Task similar to: Cohen, J.D., Barch, D.M., Carter, C.S., & Servan-Schreiber, D. (1999). Schizophrenic deficits in the processing of context: Converging evidence from three theoretically motivated cognitive tasks. *Journal of Abnormal Psychology, 108*, 120–133.
3. Visual working memory task
  - Task similar to: Luck, S. J., & Vogel, E. K. (1997). The capacity of visual working memory for features and conjunctions. *Nature, 390*(6657), 279–281.
